# ﻿A new eyeless species of *Nereis* (Annelida, Nereididae) from deep-sea sediments of the northern South China Sea

**DOI:** 10.3897/zookeys.1134.94198

**Published:** 2022-12-05

**Authors:** Jun-Hui Lin, Ya-Qin Huang, Qian-Yong Liang, Xue-Bao He

**Affiliations:** 1 Third Institute of Oceanography, Ministry of Natural Resources, 178 Daxue Road, Xiamen 361005, China Third Institute of Oceanography, Ministry of Natural Resources Xiamen China; 2 MLR Key Laboratory of Marine Mineral Resources, Guangzhou Marine Geological Survey, China Geological Survey, Guangzhou 510070, China Guangzhou Marine Geological Survey, China Geological Survey Guangzhou China

**Keywords:** Nereidiformia, phylogeny, polychaete, systematics, taxonomy

## Abstract

A variety of nereidid species have been reported from the South China Sea, although little is known about the deep-sea species in this area. Recently, two specimens belonging to a novel nereidid polychaete were collected from a sedimentary habitat during an environmental survey to a deep-sea basin where cold seeps occur. This new species, *Nereistricirrata***sp. nov.**, is described herein, based on morphological and molecular analyses. The most noteworthy feature is the absence of eyes on the prostomium; it can be distinguished from other eyeless *Nereis* species by the arrangement of conical paragnaths on the pharynx, the nature of homogomph falcigers and the shape of notopodial lobes in posterior chaetigers. The reconstructed phylogenetic tree, using concatenated sequences of mtCOI, 16S, and 18S rRNA, showed that all *Nereis* species included in this study form a monophyletic clade with full support. The mtCOI-based interspecific comparisons revealed a high genetic divergence (23.1%–37.3% K2P) from four-eyed *Nereis* species with the available sequences. This is the first record of an eyeless *Nereis* species in the South China Sea.

## ﻿Introduction

Members of the annelid family Nereididae are commonly seen in marine and brackish benthic communities. The family is among the most diverse taxa groups, with 709 nominal species in 43 genera (Read and Fauchald 2021) distributed from the intertidal to the abyss (Wilson 2000). Nereidids are well represented in the deep sea at depths greater than 2000 m (Paterson et al. 2009). To date, a large number of deep-sea nereidid species have been recorded in previous surveys conducted in areas off New England to Bermuda (Hartman and Fauchald 1971), off western Mexico, east Pacific (Fauchald 1972), off the Japanese Pacific (Imajima 2009), and in the vicinity of eastern Pacific vents (Blake 1985; Blake and Hilbig 1990). Interestingly, some of these species lack eyes on the prostomium or have a sunken depression in place where the eyes usually occur (Blake 1985). These eyeless species have been assigned to a variety of nereidid genera, such as *Ceratocephale* (Hutchings and Reid 1990; Böggemann 2009), *Micronereides* (Day 1963), *Neanthes* (Kirkegaard 1995; Shimabukuro et al. 2017), *Nereis* (Fauchald 1972; Blake 1985; Blake and Hilbig 1990; Imajima 2009), *Nicon* (Fauchald 1972), *Rullierinereis* (Böggemann 2009; Imajima 2009), *Tambalagamia* (Shen and Wu 1993), and *Typhlonereis* (Bakken 2003), with *Nereis* species being the richest in species number. *Nereis* Linnaeus, 1758, is the type genus of the family Nereididae with more than 300 described species around the world, characterized by the presence of conical paragnaths in both pharyngeal rings and homogomph falcigers in the posterior notopodia (Sun and Yang 2004).

The South China Sea (SCS) is the largest marginal sea in the western Pacific, a biogeographic region which harbors diverse marine fauna (Salazar-Vallejo et al. 2014). Quite a few polychaete species in the family Nereididae have been reported from this area (Gallardo 1968; Sun and Yang 2004). Recently, Glasby et al. (2016) compiled a list of annelid species (excluding clitellates and siboglinids) from separate taxonomic publications and prepared a catalogue of polychaete fauna recorded in the South China Sea. In this species list, 1257 species in 73 families were reported from this area, with Nereididae being the most well-studied and diverse annelid family consisting of 134 species. These nereidid species are mostly recorded from shallow water, whereas little is known about the deep-sea species in this area owing to the difficulty in collecting specimens.

During an environmental survey to a deep-sea basin of the northern South China Sea in 2019, where cold seeps occur, two interesting nereidid specimens without prostomial eyes were collected from a sedimentary habitat. In this study, they are described and illustrated as a new species, *Nereistricirrata* sp. nov., based on morphological and molecular analyses. This is the first record of an eyeless *Nereis* species in the South China Sea.

## ﻿Materials and methods

### ﻿Field sampling

**Figure 1.**
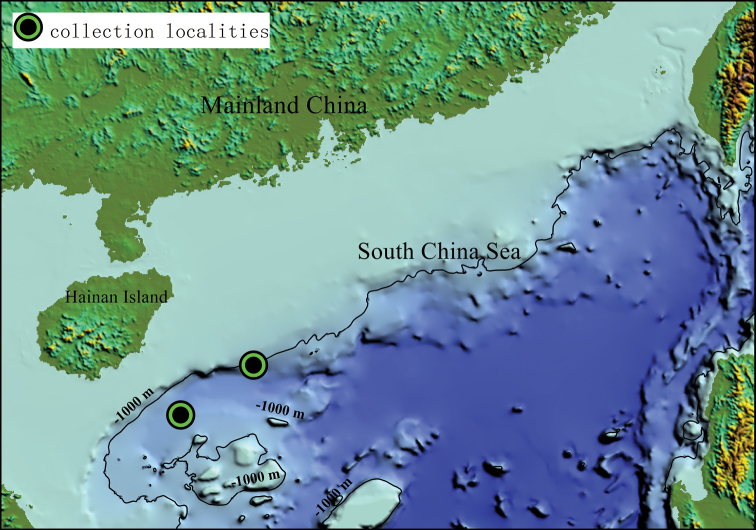
Map showing the two collection localities in the South China Sea.

In June 2019, sediment samples were collected at two sites in a deep-sea basin of the northern South China Sea (Fig. 1) using a box sampler onboard the R/V ‘Haiyangdizhi 10’. Subsequently, the sediment samples were washed through a 0.25 mm sieve with chilled, filtered seawater (4 °C) on board. The fauna retained by the sieve were fixed in either 95% ethanol or 8% diluted formalin. One of these specimens was complete, but broken into two fragments. For the complete specimen, chaetigers of the posterior fragment were dissected in the field and then preserved in 95% ethanol. The anterior fragment and the remaining posterior fragment were preserved in 8% diluted formalin in seawater.

### ﻿Morphological observations

In the laboratory, the specimens were examined using a Leica MZ9.5 optical stereoscope and a Leica DM6B compound microscope. Several parapodia from anterior, middle, and posterior parts of the holotype were dissected and mounted on slides for observation. Light photographs were taken under a Leica M205A stereoscope, equipped with a DFC 550 digital camera. The shape of the chaetae was observed and photographed under a Leica compound microscope (DM6B). Plates were prepared using the software Adobe Photoshop CS5. The terminology of parapodial structures used in this study follows Bakken and Wilson (2005) and Villalobos-Guerrero and Bakken (2018). The type material examined in this study was deposited at the Third Institute of Oceanography, Ministry of Natural Resources, Xiamen, China (**TIO, MNR**).

### ﻿Molecular analysis

The total genomic DNA was extracted from the ethanol-preserved tissue sample of the holotype using a Transgen Micro Genomic DNA EE 181 Kit (Transgen, Beijing, China), following the manufacturer’s protocol. Polymerase chain reactions (PCRs) were conducted to amplify partial sequences of mitochondrial (mtCOI, 16S) and nuclear (18S, H3) genes using primer sets as shown in Table 1. The PCR mixtures contained 10 μl of TakaRa 10× Ex Taq buffer, 8 μl of dNTP mixture (2.5 mM), 2 μl of each primer (10 μM), 0.5 μl of TakaRa Ex Taq (5 U/μl), and 4 μl of DNA template and deionized water was added to make up a final volume of 100 μl. The thermal cycling conditions were as follows: 95 °C/240s – (95 °C/45s – 42 °C/60s – 72 °C/80s) *35 cycles – 72 °C/420s for mtCOI and 16S; 95 °C/240s – (95 °C/45s – 45 °C/60s – 72 °C/80s) *35 cycles – 72 °C/420s for 18S1, 18S2, 18S3, H3. The resulting PCR products were checked using 1% agarose gel electrophoresis and the successful PCR products were purified using a Transgen Quick Gel Extraction EG 101 Kit (Transgen, Beijing, China), following the manufacturer’s protocol. Sequencing of the purified DNA samples was performed on an ABI 3730XL DNA Analyzer (Applied Biosystems) at Biosune Company (Xiamen, China). Obtained sequences (18S1, 18S2 and 18S3) were manually assembled into a consensus sequence using the software DNAMAN 8 (Lynnon Biosoft, Quebec, Canada), then checked for potential contamination using BLAST. Eventually, about 649 bp of COI, 437 bp of 16S, 1330 bp of 18S, and 308 bp of H3 were successfully amplified in this study.

**Table 1.** List of primer sets used for PCRs and sequencing in this study.

Gene	Primer name	Sequence (5' to 3')	Reference
COI	LCO 1490	GGTCAACAAATCATAAAGATATTGG	Folmer et al. (1994)
	HCO 2198	TAAACTTCAGGGTGACCAAAAAATCA	Folmer et al. (1994)
16S	16SarL	CGCCTGTTTAACAAAAACAT	Palumbi (1996)
	16SbrH	CCGGTCTGAACTCAGATCACGT	Palumbi (1996)
H3	aF	ATGGCTCGTACCAAGCAGAC	Colgan et al. (1998)
	aR	ATATCCTTRGGCATRATRGTGAC	Colgan et al. (1998)
18S1	F	GCTGTATGTACTGTGAAACTGCG	Song et al. (2018)
	R	GGAATTACCGCGGCTGCTGGCACC	Song et al. (2018)
18S2	F	GTTCGATTCCGGAGAGGGAGCCT	Song et al. (2018)
	R	GTTTCGGCCTTGCGACTATACTT	Song et al. (2018)
18S3	F	ACTGCGAAAGCATTTGCCAAGAGT	Song et al. (2018)
	R	CACCTACGGAAACCTTGTTACGAC	Song et al. (2018)

For phylogenetic analyses, the sequences of related genera of Nereididae were downloaded from GenBank, as well as species from Hesionidae (sister to Nereididae as verified by Dahlgren et al. 2000) as outgroups (more detail see Appendix 1). Sequences for each gene were aligned, respectively, using MUSCLE (Edgar 2004) implemented in MEGA X (Kumar et al. 2018) for COI and MAFFT (Katoh et al. 2002) for 16S and 18S with default setting. The unaligned sequences and highly divergent regions were removed using Gblocks 0.91b (Castresana 2000). SequenceMatrix v. 1.7.8 (Vaidya et al. 2011) was used to achieve a concatenated sequence of the three genes. Phylogenetic analyses were performed using the maximum likelihood (ML) and Bayesian inference (BI) methods. The ML analysis on the concatenated sequence was conducted in raxmlGUI 1.5 beta (Silvestro and Michalak 2012) using the GTR+G+I model and 1000 thorough bootstrap pseudoreplicates. The BI analysis was performed using MrBayes v. 3.2.6 (Ronquist et al. 2012), with four Markov chains run for 10 million generations, sampled every 1000 generations. The first 25% of these were discarded as burn-in. The tree was edited using FigTree v. 1.4 (Rambaut 2012) and Adobe Photoshop CS5. Interspecific comparisons were made with aligned COI sequences of *Nereis* species available in GenBank, using the Kimura’s two-parameter (K2P) model (Kimura 1980) implemented in MEGA X.

## ﻿Results

### ﻿Systematics

**Order Phyllodocida Dales, 1962**

#### Family Nereididae de Blainville, 1818

##### ﻿Genus Nereis

Taxon classificationAnimaliaPhyllodocidaNereididae

Linnaeus, 1758

47F4D53C-76ED-57F1-A9A2-2716A3AA2FD4

###### Type species.

*Nereispelagica* Linnaeus, 1758.

###### Generic diagnosis

**(after Bakken and Wilson 2005; Bakken et al. 2018).** Prostomium with entire anterior margin, one pair of antennae, one pair of biarticulated palps with conical palpostyles. Peristomium apodous, greater than length of chaetiger 1, with four pairs of tentacular cirri. Eyes present or absent. Conical paragnaths present on both maxillary and oral ring of pharynx. Notopodial dorsal ligule similar in size in anterior and posterior chaetigers or markedly reduced on posterior chaetigers. Notopodial prechaetal lobe present or absent, smaller than notopodial dorsal ligule on anterior chaetigers, usually reduced or absent posteriorly. Dorsal cirrus basally attached to notopodial dorsal ligule throughout all chaetigers, lacking basal cirrophore. Notoaciculae absent from chaetigers 1 and 2. Notochaetae: homogomph spinigers, homogomph falcigers present. Neurochaetae, dorsal fascicle: homogomph spinigers present, heterogomph falcigers on anterior chaetigers present or absent, on posterior chaetigers present. Neurochaetae, ventral fascicle: heterogomph spinigers present or absent, heterogomph falcigers present or absent.

##### ﻿ Nereis tricirrata sp. nov.

Taxon classificationAnimaliaPhyllodocidaNereididae

ABD9FED5-3FB8-5E81-9B00-DDA25FB013D4

https://zoobank.org/67AD5443-63CA-4E5E-9710-B81A4CF60349

**Figure 2.**
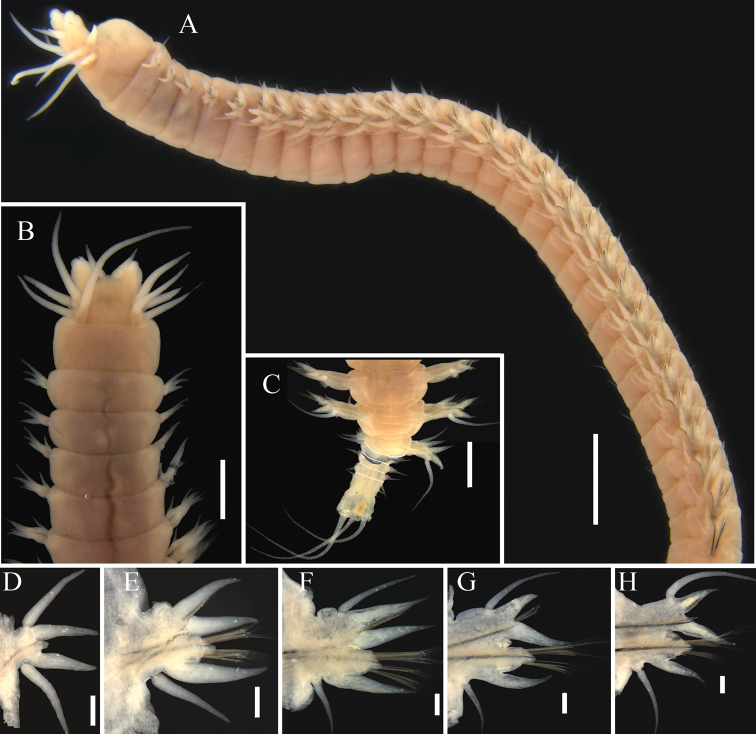
*Nereistricirrata* sp. nov., holotype (TIO-BTS-Poly-137) **A** anterior fragment, lateral view **B** anterior end, dorsal view **C** posterior end, dorsal view, intersegmental grooves of regenerated segments have been outlined with white lines **D–H** right parapodia (chaetigers 1, 5, 20, 40, posterior end), posterior view. Scale bars: 1 mm (**A–C**); 0.5 mm (**D–H**).

**Figure 3.**
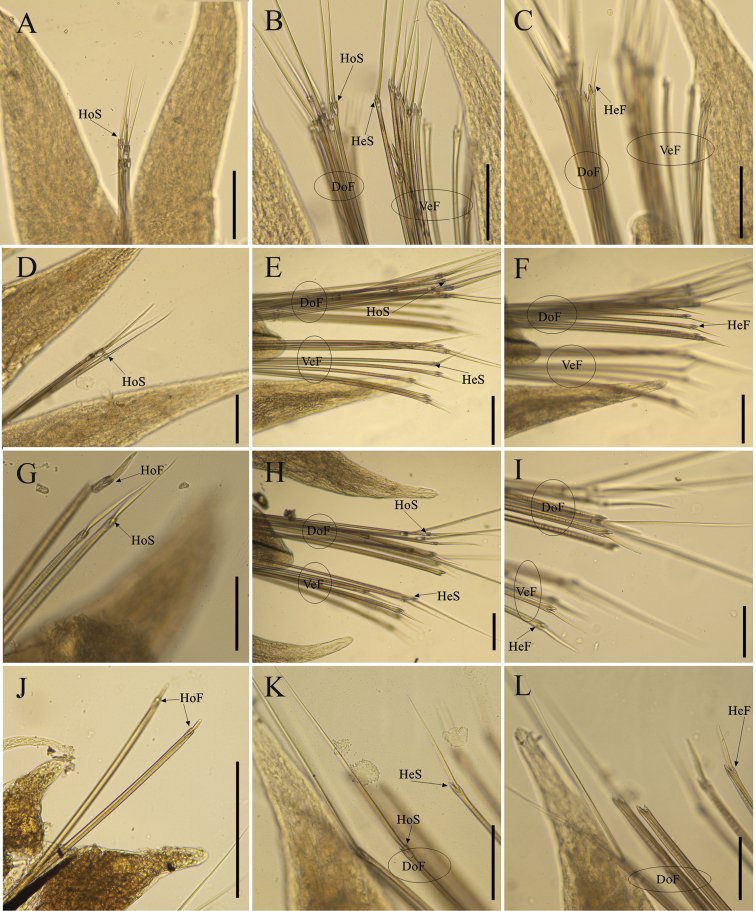
*Nereistricirrata* sp. nov., holotype and paratype **A** chaetiger 5, notochaetae **B, C** chaetiger 5, neurochaetae **D** chaetiger 20, notochaetae **E, F** chaetiger 20, neurochaetae **G** chaetiger 40, notochaetae **H, I** chaetiger 40, neurochaetae **J** posterior end, notochaetae (from paratype, as blades of notochaetae missing in the posterior fragment of holotype) **K, L** posterior end, neurochaetae. Abbreviations: HoS, homogomph spiniger; HoF, homogomph falciger; HeS, heterogomph spiniger; HeF, heterogomph falciger; DoF, dorsal fascicle; VeF, ventral fascicle. Scale bars: 100 μm (**A–L**).

**Figure 4.**
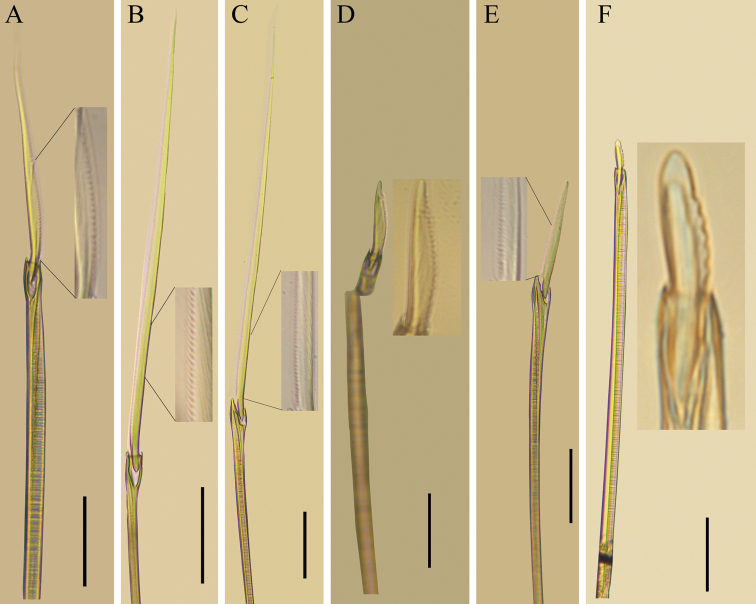
**A–E***Nereistricirrata* sp. nov. holotype (TIO-BTS-Poly-137) and **F** paratype (TIO-BTS-Poly-138) **A** notochaetae, homogomph spiniger, chaetiger 40 **B** neurochaetae, homogomph spiniger, dorsal fascicle, chaetiger 5 **C** neurochaetae, heterogomph spiniger, ventral fascicle, chaetiger 40 **D** notochaetae, homogomph falciger, chaetiger 40 **E** neurochaetae, heterogomph falciger, dorsal fascicle, chaetiger 20 **F** notochaetae, homogomph falciger, posterior parapodia. Scale bars: 50 μm (**A–F**).

Figs 2A–H , 3A–L , 4A–F

###### Material examined.

***Holotype***: TIO-BTS-Poly-137, complete, northern South China Sea, (17°33'N, 111°9'E), 1766 m depth, coll. Jun-Hui Lin, 16 June 2019. ***Paratype***: TIO-BTS-Poly-138, incomplete, northern South China Sea, (18°26'N, 112°26'E), 1157 m depth, coll. Jun-Hui Lin, 21 June 2019.

###### Sequences.

OP292645, COI gene, 649 bp; OP292646, 16S gene, 437 bp; OP292647, 18S gene, 1330 bp; OP292648, histone H3, 308 bp; extracted from ethanol-preserved tissue of the holotype.

###### Diagnosis.

The new species is characterized by: (1) absence of eyes on the prostomium; (2) possession of three anal cirri instead of two on the pygidium; (3) few paragnaths on both rings of the pharynx; (4) notopodial and neuropodial ligules acutely conical; and (5) homogomph falcigers in posterior notopodia with several coarse teeth.

###### Description.

Holotype complete but broken into two fragments. Body tapering posteriorly. Anterior fragment 35.27 mm long for 44 chaetigers, remaining posterior fragment 7.28 mm long for 15 chaetigers (including regenerated segments), maximum width 2.1 mm (excluding parapodia) at chaetiger 7. Paratype incomplete, broken into three fragments with 45 chaetigers, 12 chaetigers and 7 chaetigers, respectively. Body in formalin light brown. Preserved specimens without pigmentation (Fig. 2A).

Prostomium pentagonal and slightly longer than wide, with one pair of digitiform frontal antennae (Fig. 2B). One pair of biarticulated palps arising antero-laterally, palpophores cylindrical, palpostyles globular. Eyes absent (Fig. 2B).

Peristomium apodous, 1.5 times as long as chaetiger 1. Four pairs of tentacular cirri slender, distally tapered (Fig. 2B); postero-dorsal pair the longest, extending to chaetiger 3.

Pharynx dissected, with dark brown jaws, distally curved, each with 15 blunt teeth on cutting edge. Small conical paragnaths sparse on both rings, arranged as follows: Area I = 0; II = 4 cones in a row; III = 0; IV = 2; V = 0; VI = 1; VII-VII = 2.

First two chaetigers uniramous, remaining ones biramous. Uniramous chaetigers with acutely conical dorsal ligules, subequal in length and of similar shape to ventral ligule (Fig. 2D). Dorsal cirri slightly longer than dorsal ligules.

Notopodia of biramous chaetigers with dorsal and ventral ligules, without notopodial prechaetal lobes. Notopodial dorsal ligules acutely conical (Fig. 2E–H), gradually becoming reduced towards posterior end (Fig. 2F–H). Dorsal cirri slender and attached to base of dorsal ligule throughout, subequal in length to notopodial dorsal ligules in anterior parapodia (Fig. 2D), and markedly longer than dorsal ligules in middle and posterior parapodia (Fig. 2G, H). Notopodial ventral ligules acutely conical, subequal in length to dorsal ligules in anterior parapodia (Fig. 2E), and 1.5–2 times length of dorsal ligules in posterior parapodia (Fig. 2G, H).

Neuropodia of biramous chaetigers with neuroacicular ligules subtriangular, postchaetal lobes rounded (Fig. 2E–H). Neuropodial ventral ligules acutely conical (Fig. 2E), longer than neuroacicular ones, decreasing in size to posterior end (Fig. 2E–H). Ventral cirri attached to ventral edge of parapodia, conical in anterior parapodia, becoming slender and cirriform from middle parapodia (Fig. 2F–H). Ventral cirri shorter than neuropodial ventral ligules in most chaetigers, but longer in chaetigers near pygidium (Fig. 2H).

In anterior parapodia, notochaetae with four homogomph spinigers (Fig. 3A, D); neurochaetae homogomph spinigers and heterogomph falcigers in dorsal fascicles (Fig. 3B, C, E, F), heterogomph spinigers and falcigers in ventral fascicles (Fig. 3B, E). In mid-body, notochaetae with two homogomph spinigers and one homogomph falciger (Fig. 3G); neurochaetae as in anterior parapodia (Fig. 3H, I). In posterior parapodia, notochaetae with two homogomph falcigers (Fig. 3J); neurochaetae as in anterior parapodia (Fig. 3K, L). Neurochaetae decreasing gradually in number towards posterior end.

All spinigers with long blades finely serrated (Fig. 4A–C); blade of notopodial spinigers shorter, but thicker than neuropodial ones. Notopodial falcigers commencing between chaetigers 20–30 (chaetiger 24 in paratype), with straight, finely serrated, blunt-tipped blade in mid-body (Fig. 4D), but with coarse teeth on relatively short blade in posterior parapodia (Fig. 4F). Neuropodial falcigers with relatively long, serrated, and blunt-tipped blade (Fig. 4E).

Posterior end with six or seven regenerated chaetigers (Fig. 2C), which are disproportionately smaller than normal chaetigers. Pygidium with three anal cirri, all filiform, one on mid-dorsal and one on each of the ventro-lateral sides (Fig. 2C).

###### Etymology.

The specific epithet *tricirrata* is composed by the Latin prefix *tri*-, meaning three, and the Latin noun *cirrus*, and refers to the three anal cirri present on the pygidium, one on the mid-dorsal and one on each of the ventro-lateral sides.

###### Distribution.

Currently only known from the deep-sea sedimentary habitat in the northern South China Sea.

###### Habitat.

Deep-sea soft sediments characterized by foraminiferal ooze at depths between 1100 m and 1800 m.

###### Phylogenetic analysis.

**Figure 5.**
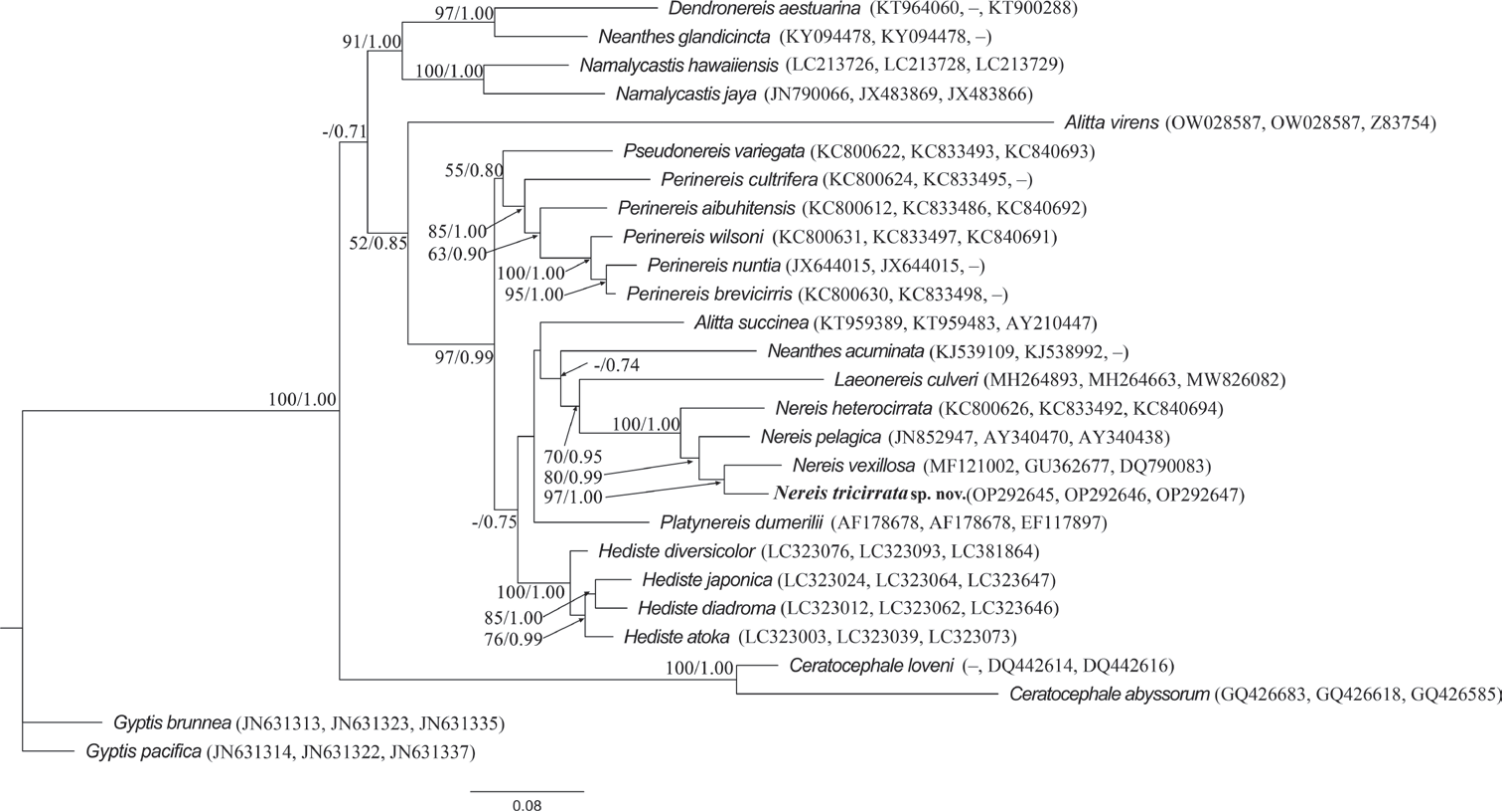
The maximum likelihood (ML) tree inferred from the concatenated sequences of three genes (mtCOI, 16S and 18S rRNA) with GenBank accession numbers. Bootstrap values and posterior probabilities values at nodes were calculated from the ML and Bayesian inference (BI) analyses, respectively. Only bootstrap values ≥ 50 and posterior probabilities ≥ 0.7 are shown. GenBank accession numbers in parenthesis are present in the order of COI, 16S, and 18S; missing markers are denoted by a dash (–).

There are no identical sequence matches on GenBank for COI and 16S. The low 18S gene divergence (0–1.9% K2P) between *Nereistricirrata* sp. nov. and other *Nereis* species revealed their close genetic relationship, including an eyeless species, *Nereissanderi* Blake, 1985 (AM159579). The reconstructed phylogenetic tree (Fig. 5), using the maximum likelihood and Bayesian inference analyses, indicates that all *Nereis* species form a monophyletic clade with 100% nodal support and confirms the placement of *Nereistricirrata* sp. nov. within the genus *Nereis*. Currently, limited sequences of eyeless *Nereis* species are available, which hinders a better understanding of the relationship among eyeless *Nereis* species. When comparing the new species to other described *Nereis* species with COI genes available in GenBank, the mtCOI-based genetic divergence (K2P) ranged from 23.1% to 37.3% (Table 2), which was comparable to that of previous studies on other nereidid genera, such as *Alitta* species (Villalobos-Guerrero and Carrera-Parra 2015), *Neanthes* species (Shimabukuro et al. 2017), and cryptic species of *Nereisdenhamensis* (Glasby et al. 2013).

**Table 2.** The mtCOI-based genetic divergence (K2P) between described *Nereis* species with the available sequences.

	Taxa	Locality	1	2	3	4	5	6	7	8	9	10	11
1	*N.multignatha* MT712473	China											
2	*N.pelagica* HQ023592	Canada	0.286										
3	*N.vexillosa* HM473512	Canada	0.285	0.259									
4	*N.zonata* HQ024404	Canada	0.262	0.238	0.284								
5	*N.denhamensis* JX294511	Australia	0.313	0.336	0.302	0.335							
6	*N.falsa* KR916890	Portugal	0.344	0.282	0.330	0.339	0.360						
7	*N.heterocirrata* MN256589	China	0.291	0.263	0.304	0.317	0.309	0.343					
8	*N.eakini* MN138408	USA	0.238	0.242	0.250	0.272	0.343	0.338	0.314				
9	*N.riisei* JF293304	Colombia	0.304	0.294	0.312	0.262	0.351	0.313	0.291	0.324			
10	*N.heronensis* JX392066	Australia	0.287	0.248	0.311	0.306	0.332	0.364	0.302	0.319	0.336		
11	*N.lizardensis* JX392060	Australia	0.290	0.307	0.296	0.306	0.277	0.320	0.291	0.287	0.303	0.327	
**12**	***N.tricirrata* sp. nov. OP292645**	SCS	0.288	0.231	0.254	0.275	0.337	0.359	0.308	0.267	0.373	0.344	0.314

###### Remarks.

*Nereistricirrata* sp. nov. is distinguished from most *Nereis* species around the world by the absence of eyes on the prostomium. With the new species in this study, seven other described *Nereis* species from the deep Pacific also lack prostomial eyes. Six of these species belong to a distinct group with greatly prolonged notopodia in the posterior parapodia, including *N.profundi* Kirkegaard, 1956, *N.anoculis* Hartman, 1960, *N.anoculopsis* Fauchald, 1972, *N.sandersi* Blake, 1985, *N.piscesae* Blake & Hilbig, 1990, and *N.abyssa* Imajima, 1990. Comparison of the two eyeless species bearing normal notopodia throughout the body showed that *Nereistricirrata* sp. nov. differs from *Nereisizukai* Okuda, 1939 (Imajima 1996) from the Japanese Pacific in the arrangement of paragnaths on the pharynx and the nature of notopodial falcigers in the posterior parapodia. *Nereisizukai* possesses far denser paragnaths on the pharynx (Area I = 11; II = 52–56; III = ~ 70; IV = 50–60; V = 0; VI = 6–12; VII-VIII = ~ 62), and its notopodial falcigers lack coarse teeth on the cutting edge in the posterior parapodia. A not-formally-named *Nereis* species without prostomial eyes, labelled as *Nereis* sp. B, was recorded from off eastern Taiwan Island at depths of 2233–2551 m (Hsueh 2020). It was unclear whether *Nereis* sp. B possessed prolonged notopodia in the posterior parapodia as it was incomplete and lacked the posterior end. Despite this, *Nereis* sp. B is distinct from *Nereistricirrata* sp. nov. in that the former possesses more paragnaths than the latter (Area I = 2; II = 21; III = 37; IV = 11–30; V = 0; VI = 5–7; VII-VIII = 74). Finally, it should be noted that the new species bears three slender anal cirri on the pygidium instead of two as usually occurs in nereidid species.

## Supplementary Material

XML Treatment for Nereis

XML Treatment for Nereis tricirrata

## ﻿Appendix 1

**Table A1.** DNA sequences with GenBank accession numbers used for the phylogenetic analysis; new sequences in bold.

Species	Localities	Voucher/isolate	CO1	16S	18S	References
*Alittasuccinea*	USA	USNM: lZ: 1286800	KT959389	KT959483	AY210447*	18S from Passamaneck and Halanych 2006
*Alittavirens*	UK (COI, 16S); France (18S)	–	OW028587	OW028587	Z83754*	18S from Kim et al. 1996
*Ceratocephaleabyssorum*	Abyssal SE Atlantic	–	GQ426683	GQ426618	GQ426585	Böggemann 2009
*Ceratocephale* loveni	Sweden	SMNH 83517	–	DQ442614	DQ442616	Ruta et al. 2007
*Dendronereisaestuarina*	India	–	KT964060	–	KT900288	Direct submission
*Hedisteatoka*	Japan	–	LC323003	LC323039	LC323073	Tosuji et al. 2019
*Hedistediadroma*	Japan	–	LC323012	LC323062	LC323646	Tosuji et al. 2019
*Hedistediversicolor*	Japan	–	LC323076	LC323093	LC381864	Tosuji et al. 2019
*Hedistejaponica*	Japan	–	LC323024	LC323064	LC323647	Tosuji et al. 2019
*Laeonereisculveri*	Brazil	–	MH264893	MH264663	MW826082	Direct submission
*Namalycastishawaiiensis*	Narashino, Japan	isolate 35–1	LC213726	LC213728	LC213729	Abe et al. 2017
*Namalycastisjaya*	Kerala, India	AQJ3	JN790066	JX483869	JX483866	Magesh et al. 2012
*Neanthesacuminata*	California, USA	isolate RLF3	KJ539109	KJ538992	–	Reish et al. 2014
*Neanthesglandicincta*	Xiamen, China	497	KY094478	KY094478	–	Lin et al. 2017
*Nereisheterocirrata*	China	–	KC800626	KC833492	KC840694	Direct submission
*Nereispelagica*	Sweden	SMNH118992; SMNH 75831	JN852947	AY340470	AY340438	Norlinder et al. 2012
***Nereistricirrata* sp. nov.**	deep SCS	TIO–BTS–Poly–137	**OP292645**	**OP292646**	**OP292647**	This study
*Nereisvexillosa*	Alaska (COI); China (16S); Germany (18S)	–	MF121002	GU362677	DQ790083	Direct submission
*Perinereisaibuhitensis*	China	–	KC800612	KC833486	KC840692	Direct submission
*Perinereisbrevicirris*	China	–	KC800630	KC833498	–	Direct submission
*Perinereiscultrifera*	China	–	KC800624	KC833495	–	Direct submission
*Perinereisnuntia*	Korea	–	JX644015	JX644015	–	Won et al. 2013
*Perinereiswilsoni*	China	–	KC800631	KC833497	KC840691	Direct submission
*Platynereisdumerilii*	USA (COI & 16S); UK (18S)	–	AF178678	AF178678	EF117897	COI & 16S from Boore and Brown 2000; 18S Hui et al. 2007
*Pseudonereisvariegata*	China	–	KC800622	KC833493	KC840693	Direct submission
*Gyptispacifica*	Japan	SIO–BIC A2516, A2517	JN631314	JN631322	JN631337	Pleijel et al. 2012
*Gyptisbrunnea*	California, USA	FP collection	JN631313	JN631323	JN631335	Pleijel et al. 2012
